# Supplementary material for: Ent-homocyclopiamine B, a Prenylated Indole Alkaloid of Biogenetic Interest from the Endophytic Fungus Penicillium concentricum
Source: Molecules. 2019 Jan 9;24(2):218. doi: 10.3390/molecules24020218 (PMC6359132; doi:10.3390/molecules24020218)
Supplement: Supplementary file 1 [file molecules-24-00218-s001.pdf]

Ent-homocyclopiamine B, a prenylated indole alkaloid of biogenetic interest from the endophytic fungus *Penicillium concentricum*

Tehane Ali [1], Tiffany M. Pham [2], Kou-San Ju [1,2,3,4] and L. Harinantenaina Rakotondraibe [1,3,4,*]

1 Division of Medicinal Chemistry and Pharmacognosy, College of Pharmacy, The Ohio State University, Columbus, OH 43210, USA; rakotondraibe.1@osu.edu
2 Department of Microbiology, College of Arts and Sciences, The Ohio State University, Columbus, OH 43210, USA;
3 Infectious Diseases Institute, The Ohio State University, Columbus, OH 43210, USA
4 Center for Applied Plant Sciences, The Ohio State University, Columbus, OH 43210, USA

Table of contents

Figure S1. HRESIMS spectrum of compound **1**

Figure S2. IR spectrum of compound **1**. Recorded as thin film at 20 ºC.

Figure S3. UV/vis spectrum of compound **1**

Figure S4. ^{1}H NMR spectrum of compound **1** in CD_3OD-*d_4* (700 MHz)

Figure S5. ^{13}C NMR spectrum of compound **1** in CD_3OD-*d_4* (175 MHz)

Figure S6. HSQC spectrum of compound **1** CD_3OD-*d_4* (700 MHz)

Figures S7a and 7b. HMBC spectrum of compound **1** in CD_3OD-*d_4* (700 MHz)

Figure S8. ^{1}H-^{1}H COSY NMR spectrum of compound **1** in CD3OD-*d4* (400 MHz)

Figure S9. Experimental CD spectrum of compound **1**

LC-MS

Figure S10. Positive ion LC-MS total ion chromatogram with detection of the protonated ions of **1** and **3** in the ethyl acetate extract of *P. concentricum* cultured on rice medium. Compounds corresponding to the loss of the nitro-radical from **1** and **3** are labeled as **1a** and **3a**, respectively.

Figure S11. Growth inhibition by 50 nmoles of kanamycin (Km) or *ent*-homocyclopiamine B (**1**) against bacterial strains on agar plates. Solvent controls did not result in zones of inhibition.

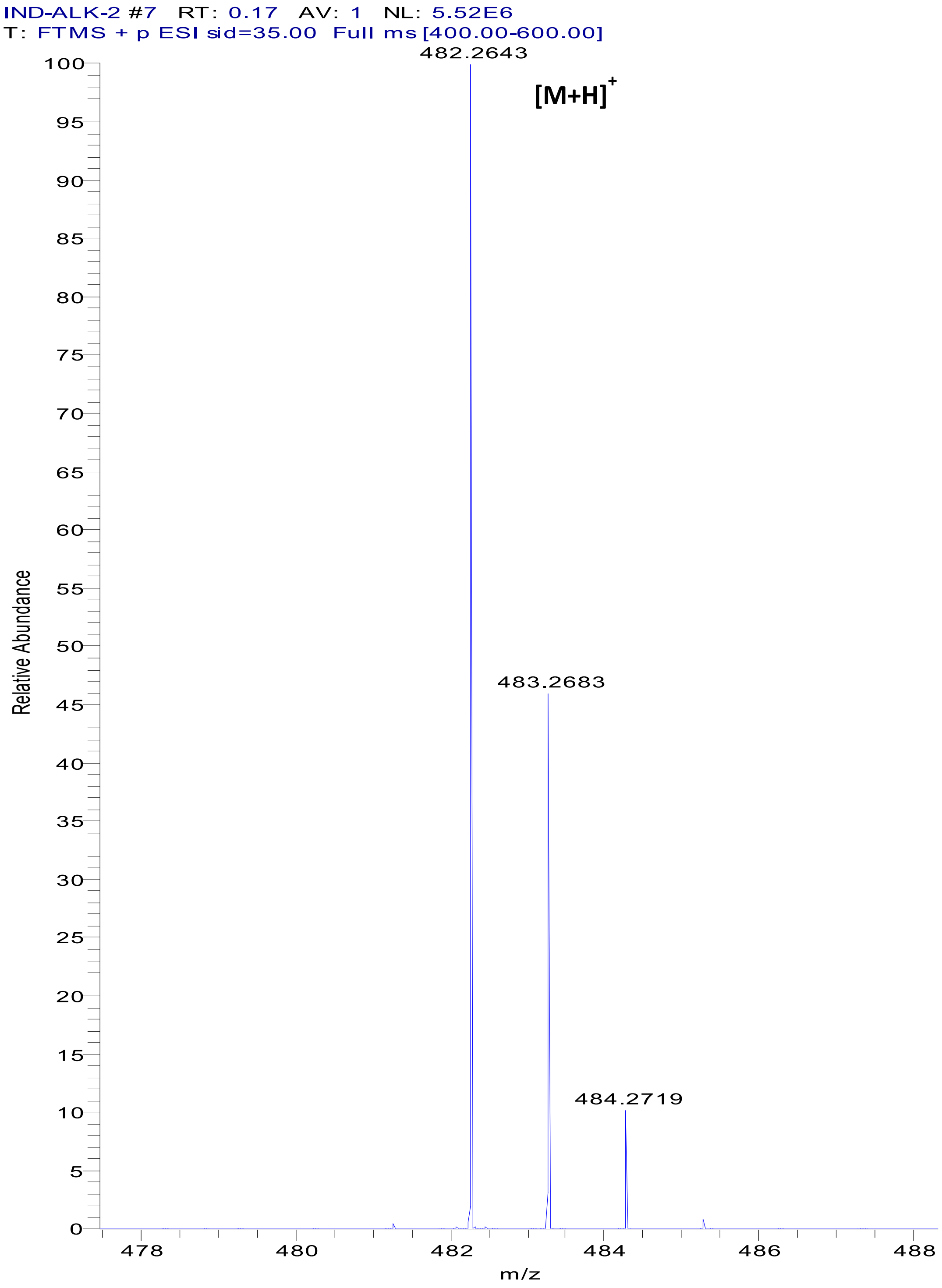

Figure S1. HRESIMS spectrum of compound **1**

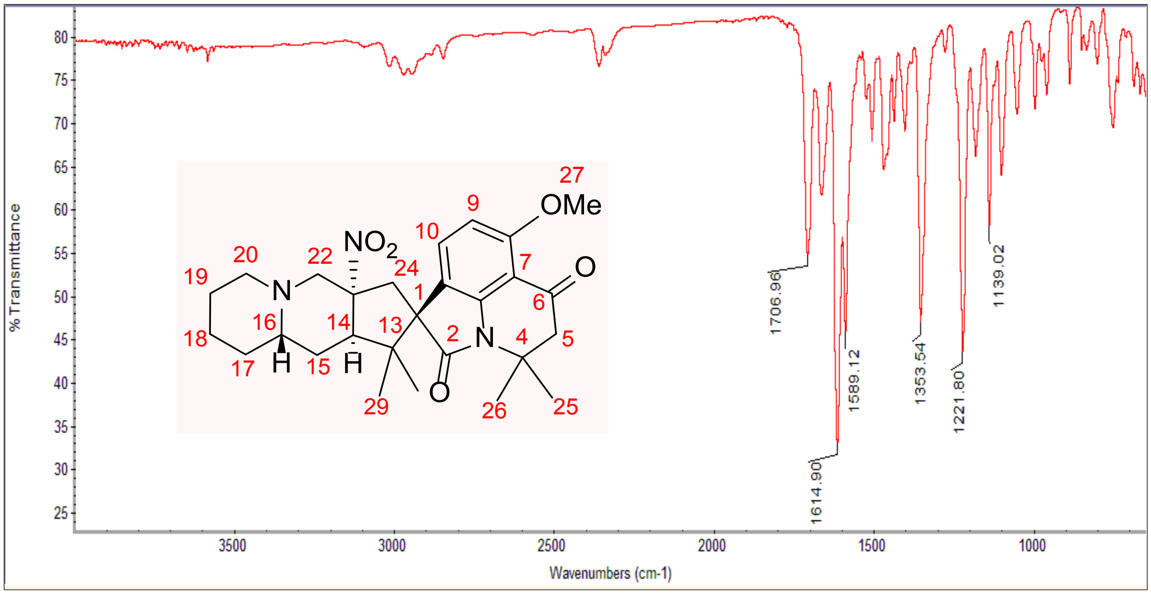

Figure S2. IR spectrum of compound **1**.

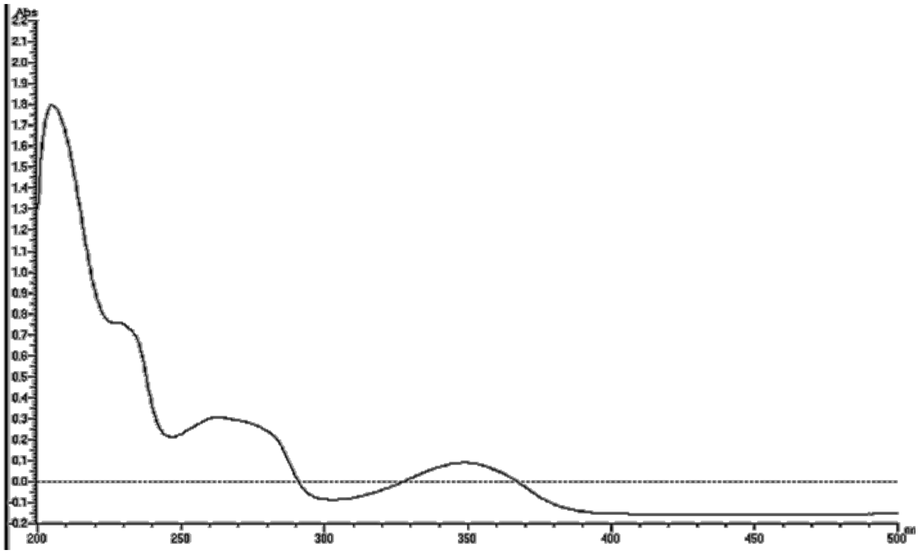

Figure S3. UV/vis spectrum of compound **1**.

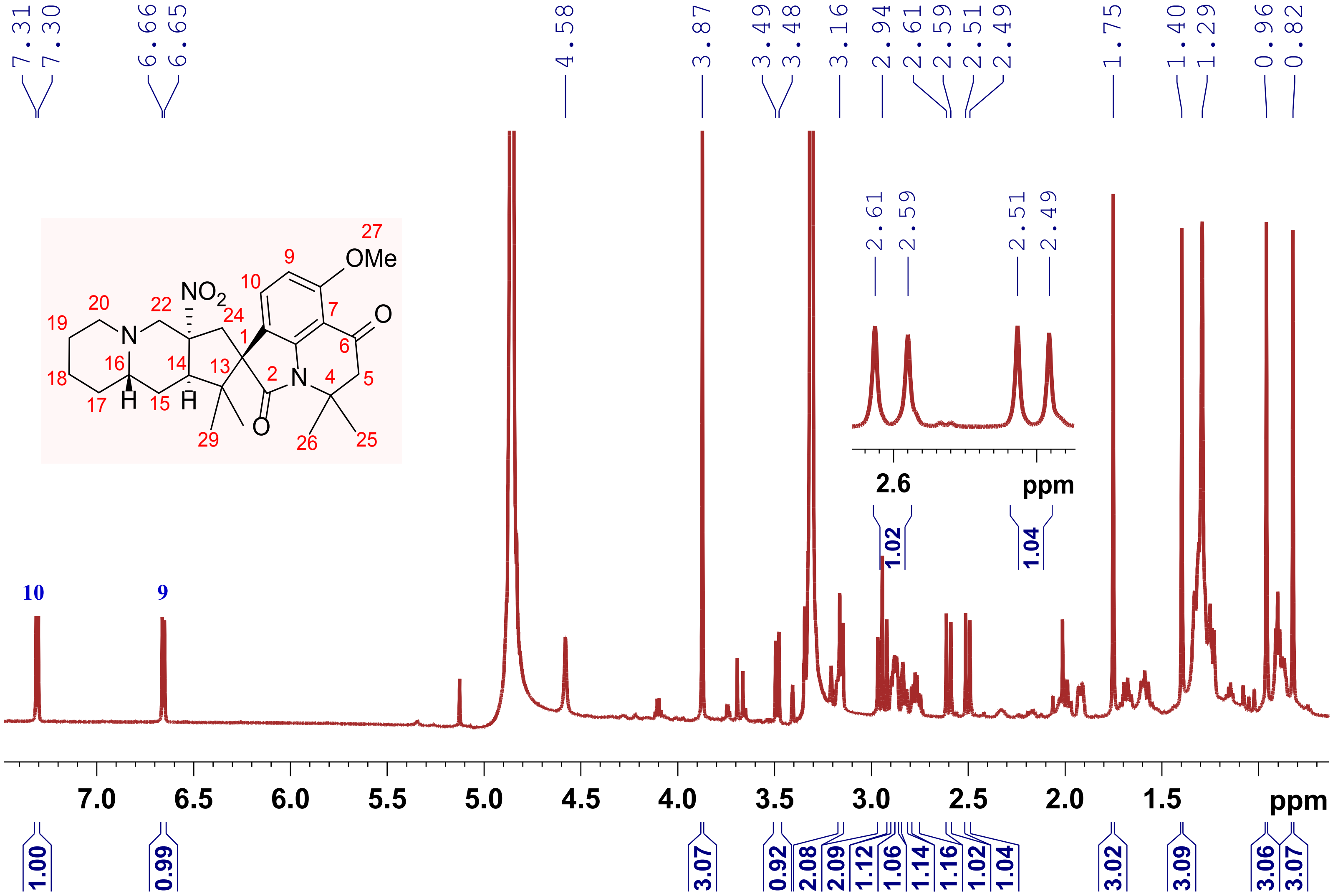

Figure S4. ^{1}H NMR spectrum of compound **1** in CD_3OD-d_4 (700 MHz)

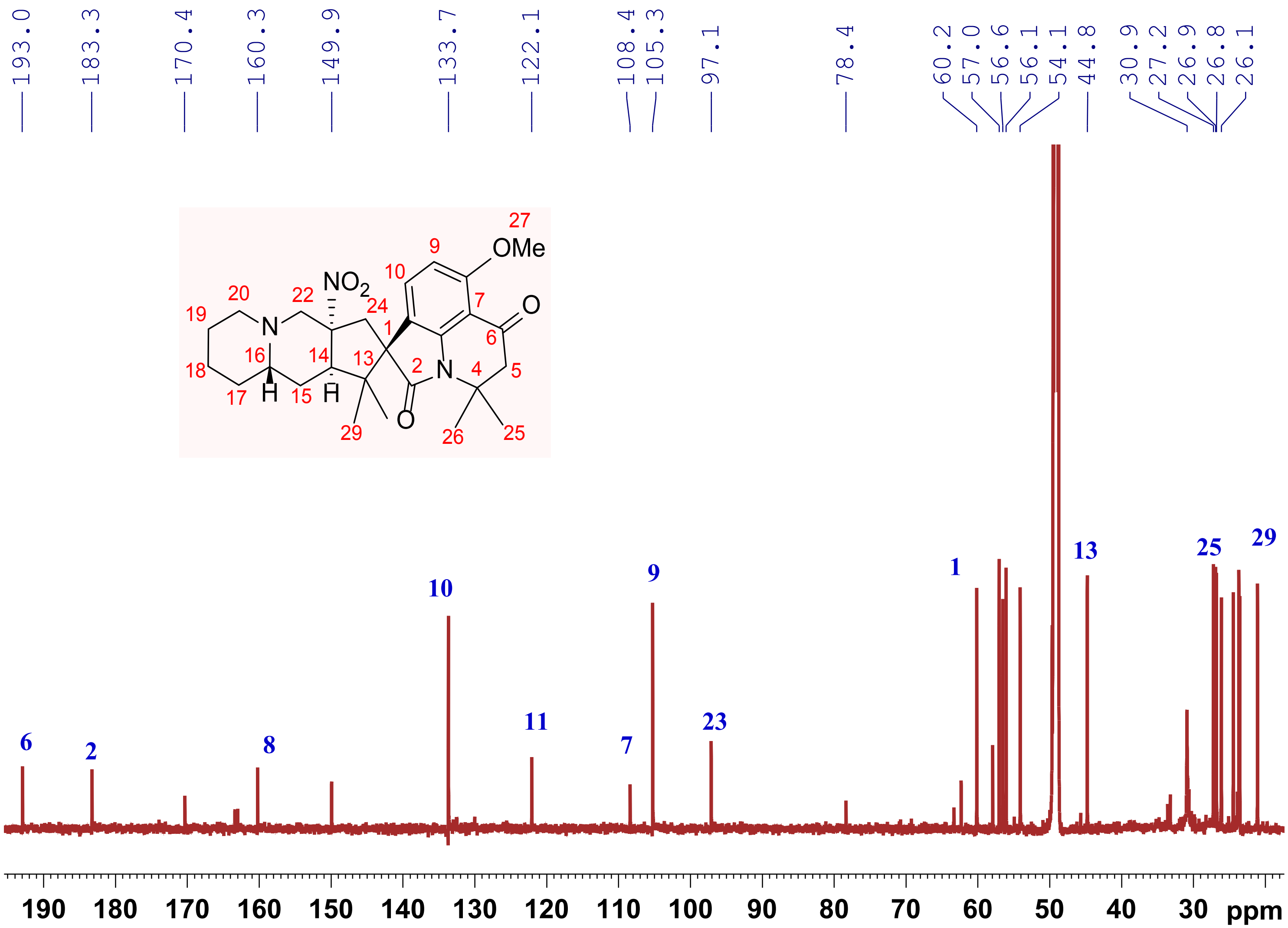

Figure S5. ^{13}C NMR spectrum of compound **1** in CD_3OD-d_4 (175 MHz)

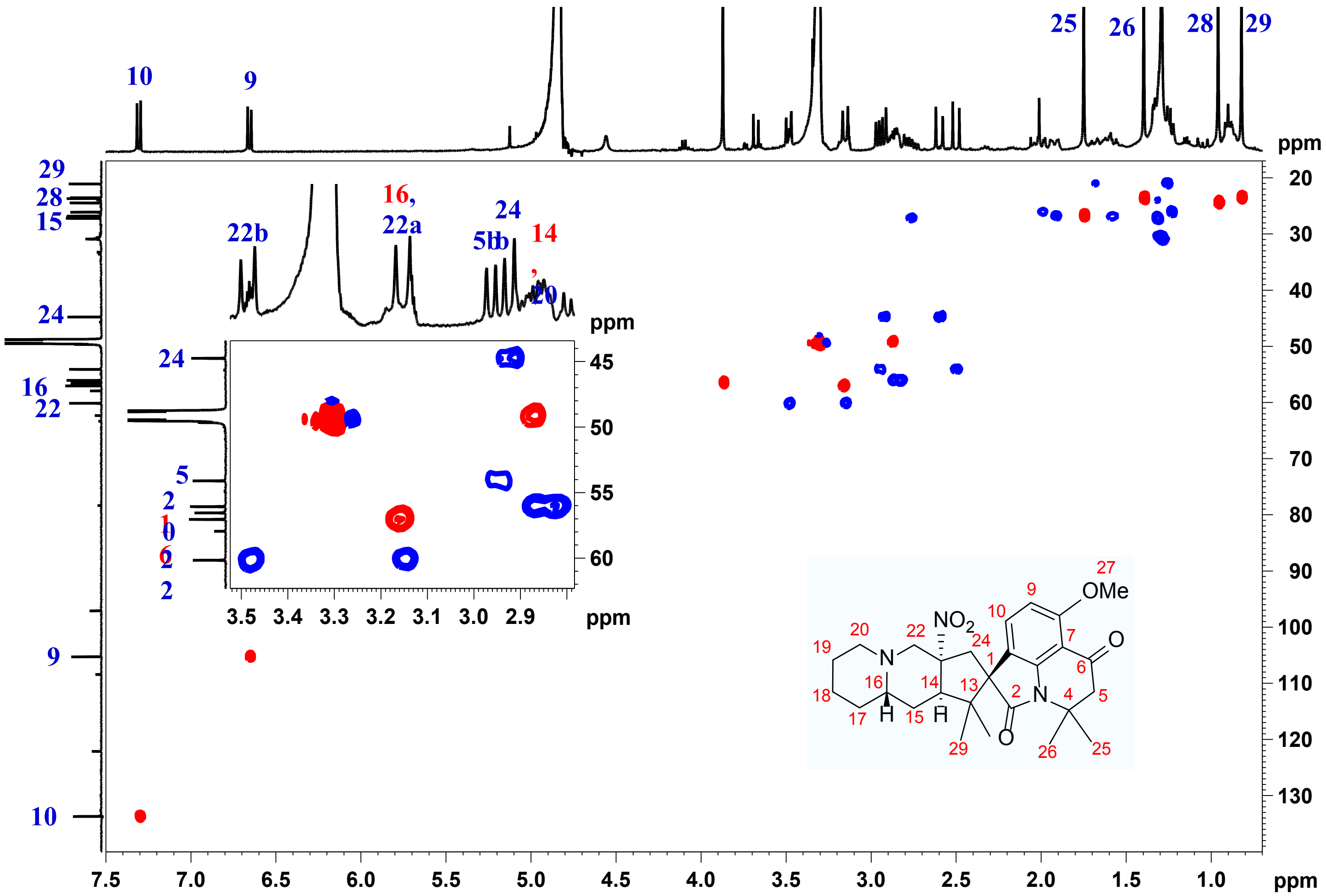

Figure S6. HSQC spectrum of compound **1** CD3OD-d_4 (700 MHz)

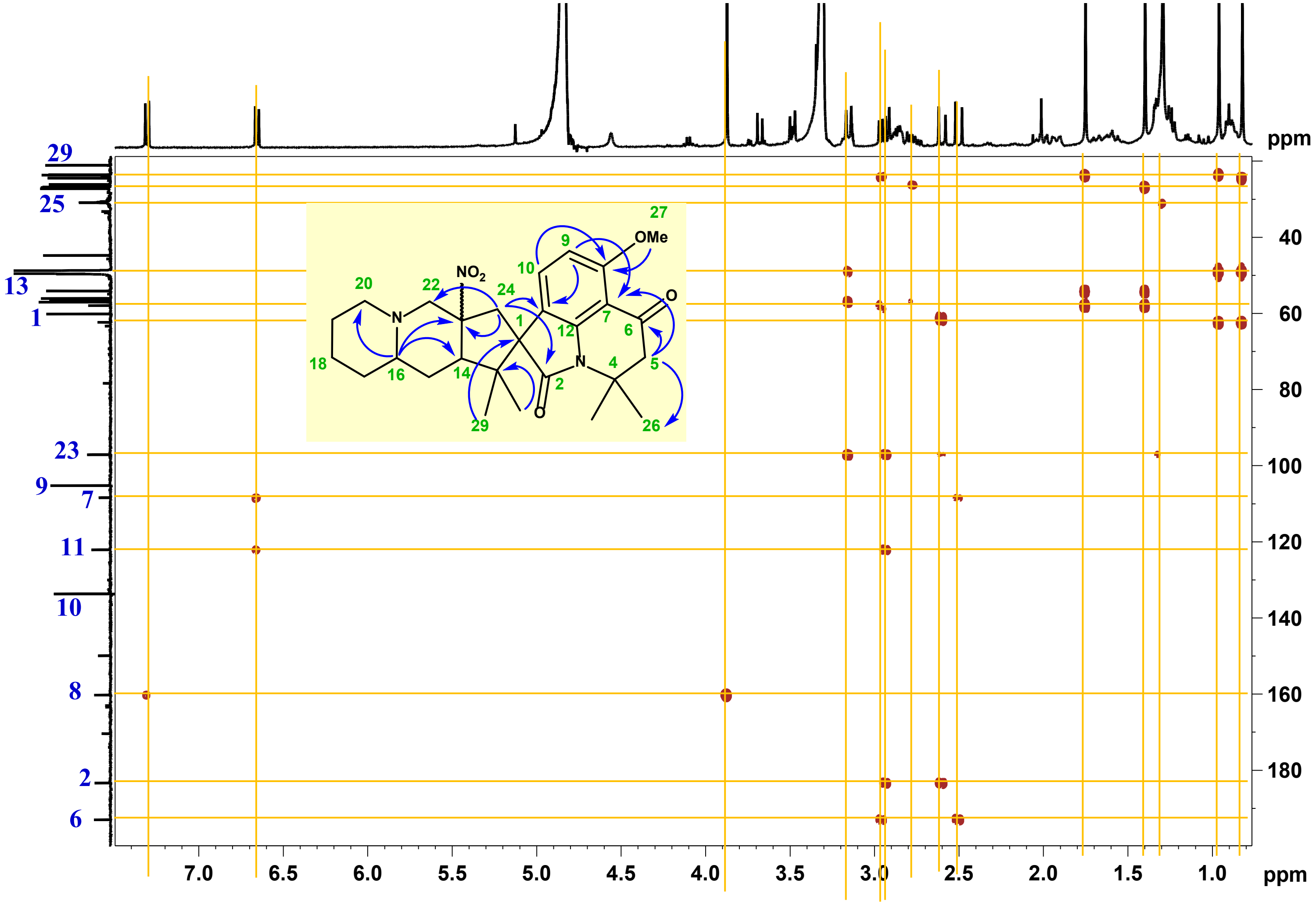

Figure S7a. HMBC NMR spectrum of **1**. Recorded in CD_3OD-d_4 at 700 MHz (^1H) and 175 MHz (^{13}C)

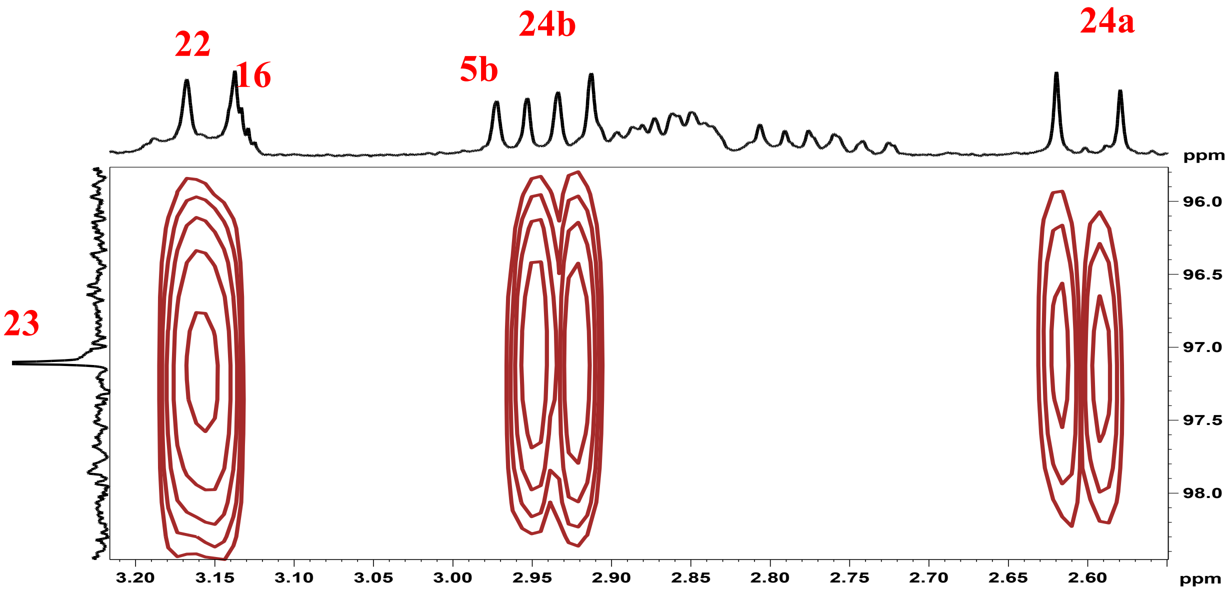

Figure S7b. Expanded HMBC NMR spectrum of **1**. Recorded in CD_3OD-d_4 at 700 MHz (^1H) and 175 MHz (^{13}C)

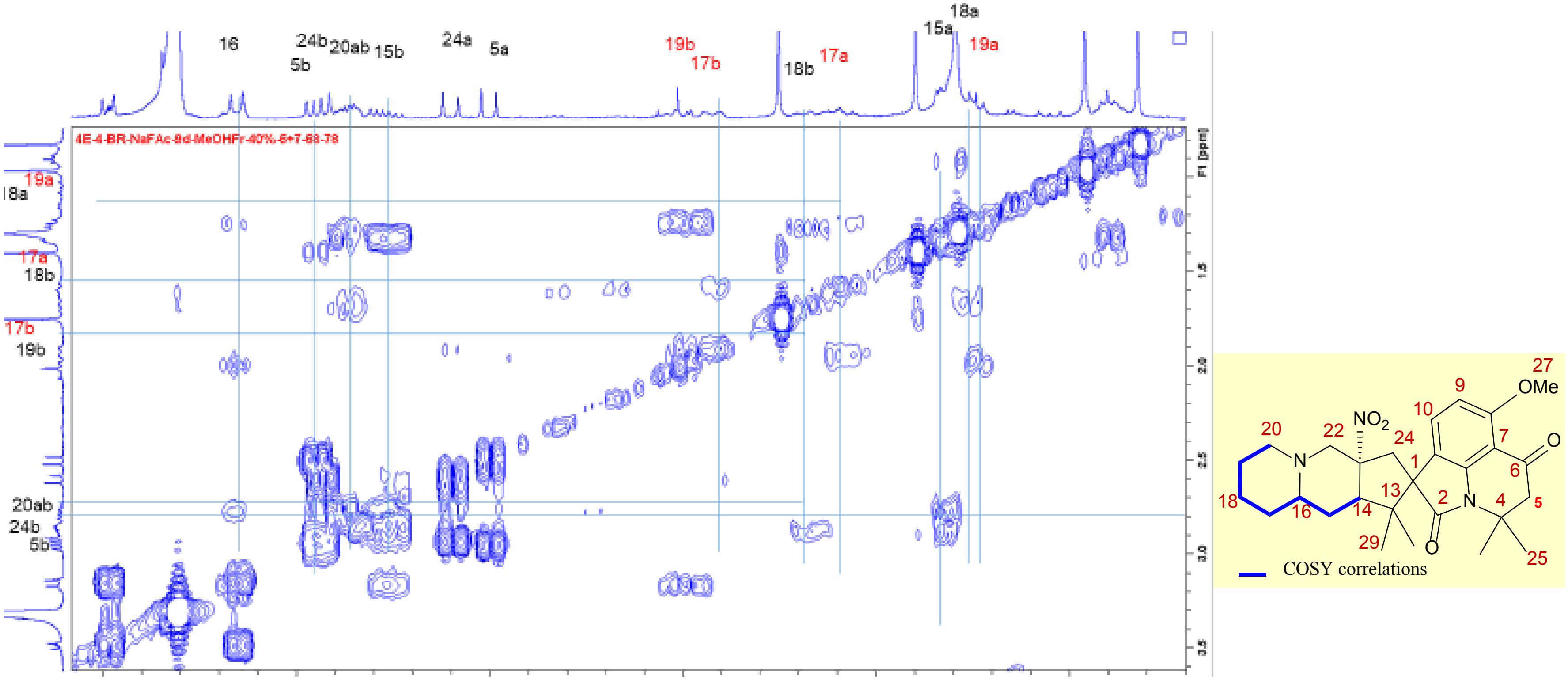

Figure S8. ^{1}H-^{1}H COSY NMR spectrum of compound **1** in CD_3OD-d_4 (400 MHz)

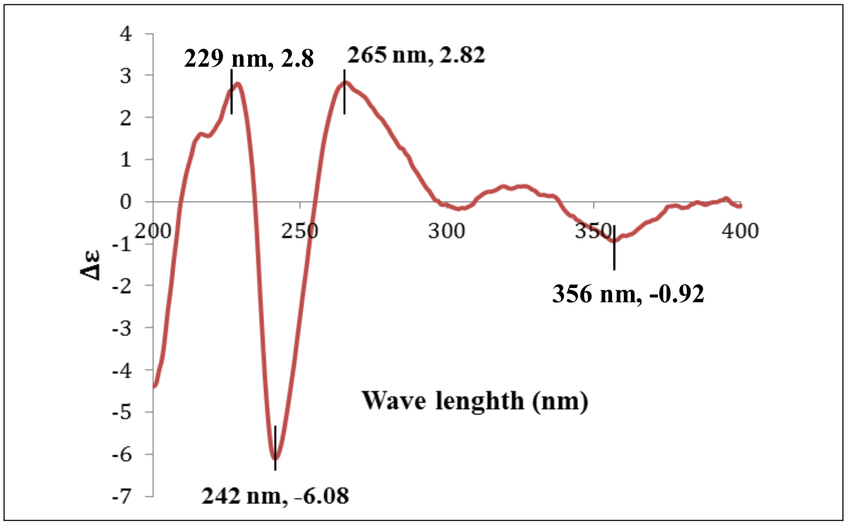

Figure S9. Experimental CD spectrum of **1**.

LC-MS

The ethyl acetate extract of *P. concentricum* grown on brown rice medium only for 11 days and the ethyl acetate extract of the fungus fermented on brown rice with supplementation of $CH_2FCOONa$ were each evaporated to dryness under vacuum. Few milligrams aliquot of each of these extract residue was dissolved in methanol and 1.5 mL of the extract was filtered using a 0.45 μm syringe filter prior to placing it into an autosampler vial.

The samples were analysed using the parameters below:

LC/MS/MS	Liquid Chromatograph: Agilent 1100<br>Mass Spectrometer: Thermo LTQ Orbitrap				
LC Column	Beckman ODS, 5μm, 4.6 mm, 25 cm<br>Part#: 235329				
Injection Vol	100 μL				
Oven Temp	25 °C				
Mobile Phase A	0.1% Formate in Water				
Mobile Phase B	0.1% Formate in Methanol				
Gradient (min)	0.0	3.0	20.0	23.0	26.0
%B	5.0	5.0	95.0	95.0	5.0
Flow Rate (mL/min)	1.0	1.0	1.0	1.0	1.0
Ion Source Parameters	Sheath Gas Flow Rate (arb) 35<br>Aux Gas Flow Rate (arb) 25<br>Sweep Gas Flow Rate (arb) 0<br>Spray Voltage (kV) 3.50<br>Temperature (°C) 275.0<br>Capillary Voltage (V) 45.0<br>Tube Lens (V) 180.0				
MS/MS Parameters	Collision Gas: Helium				
	Sheath/Aux/Ion Sweep Gas: Helium				
	Ionization Mode: Electron Spray Ionization (ESI)				
	Ion Mode: Positive				

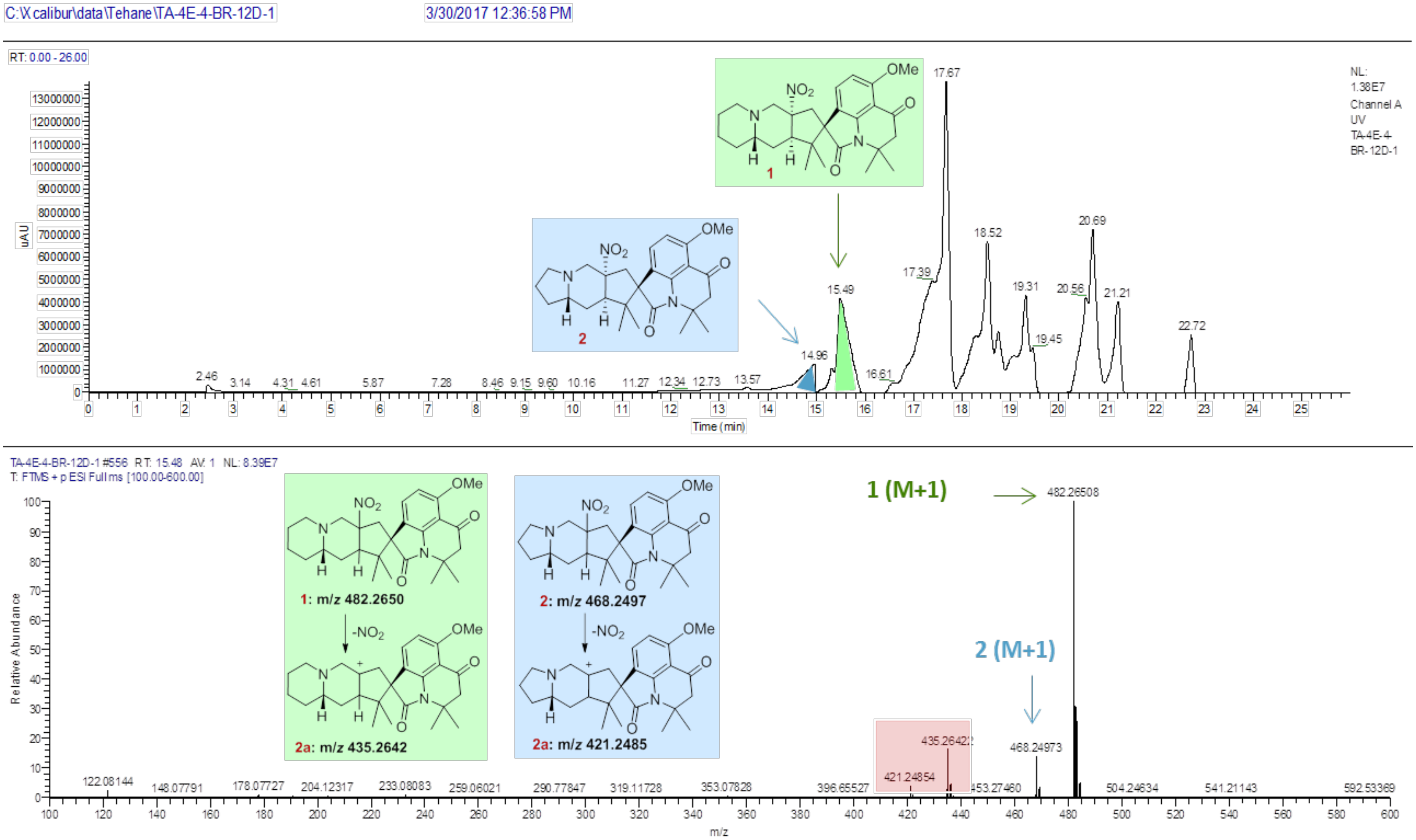

Figure S10. Positive ion LC-MS total ion chromatogram with detection of the protonated ions of 1 and 3 in the ethyl acetate extract of *P. concentricum* cultured on rice medium. Compounds corresponding to the loss of the nitro-radical from **1** and **3** are labeled as **1a** and **3a**, respectively.

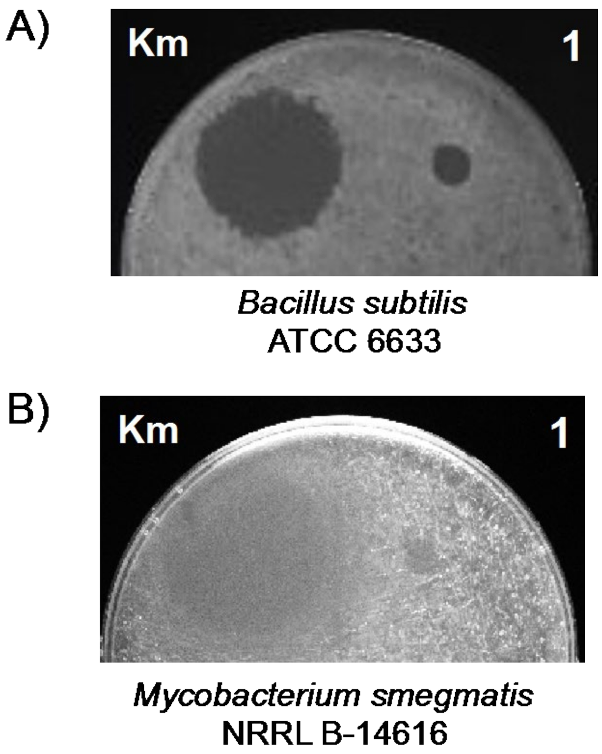

Figure S11. Growth inhibition by 50 nmoles of kanamycin (Km) or *ent*-homocyclopiamine B (**1**) against bacterial strains on agar plates. Solvent controls did not result in zones of inhibition.
